# Prognostic Factors in Patients Undergoing Physiotherapy for Chronic Low Back Pain: A Level I Systematic Review

**DOI:** 10.3390/jcm13226864

**Published:** 2024-11-14

**Authors:** Alice Baroncini, Nicola Maffulli, Marco Pilone, Gennaro Pipino, Michael Kurt Memminger, Gaetano Pappalardo, Filippo Migliorini

**Affiliations:** 1 Department of Orthopedics and Trauma Surgery, Academic Hospital of Bolzano (SABES-ASDAA), 39100 Bolzano, Italymigliorini.md@gmail.com (F.M.); 2 Department of Medicine and Psychology, University of Rome “La Sapienza”, 00185 Rome, Italy; 3 Centre for Sports and Exercise Medicine, Barts and the London School of Medicine and Dentistry, Mile End Hospital, Queen Mary University of London, London E1 4DG, UK; 4 School of Pharmacy and Bioengineering, Keele University Faculty of Medicine, Stoke on Trent ST4 7QB, UK; 5 Residency Program in Orthopedics and Traumatology, University of Milan, 20122 Milan, Italy; 6 Department of Orthopedics and Trauma Surgery, Villa Erbosa Hospital, San Raffaele University of Milan, 20132 Milan, Italy; 7 Department of Spine Surgery, Oberlinklinik GmbH, 14482 Potsdam, Germany; 8 Department of Life Sciences, Health, and Health Professions, Link Campus University, 00165 Rome, Italy

**Keywords:** low back pain, LBP, spine, physiotherapy, prognostic factors

## Abstract

**Background**: Low back pain is common. For patients with mechanic or non-specific chronic LBP (cLBP), the current guidelines suggest conservative, nonpharmacologic treatment as a first-line treatment. Among the available strategies, physiotherapy represents a common option offered to patients presenting with cLBP. The present systematic review investigates the prognostic factors of patients with mechanic or non-specific cLBP undergoing physiotherapy. **Methods**: In September 2024, the following databases were accessed: PubMed, Web of Science, Google Scholar, and Embase. All the randomised controlled trials (RCTs) which evaluated the efficacy of a physiotherapy programme in patients with LBP were accessed. All studies evaluating non-specific or mechanical LBP were included. Data concerning the following PROMs were collected: the pain scale, Roland Morris Disability Questionnaire (RMQ), and Oswestry Disability Index (ODI). A multiple linear model regression analysis was conducted using the Pearson Product–Moment Correlation Coefficient. **Results**: Data from 2773 patients were retrieved. The mean length of symptoms before the treatment was 61.2 months. **Conclusions**: Age and BMI might exert a limited influence on the outcomes of the physiotherapeutic management of cLBP. Pain and disability at baseline might represent important predictors of health-related quality of life at the six-month follow-up. Further studies on a larger population with a longer follow-up are required to validate these results.

## 1. Introduction

Low back pain (LBP) is defined as pain occurring between the lower edge of the 12th rib and the buttock creases [1]. Chronic low back pain (cLBP) is based on the persistence of pain longer than 12 weeks or 3 months [2]. The current estimates of incidence and prevalence are 0.024 to 7.0%. and 1.4 to 15.6%, respectively [3,4]. Low back pain (LBP) represents the leading cause of disability worldwide and is associated with considerable related costs for the healthcare system [5,6]. Over the past three decades, the prevalence of LBP has increased, driven by biological factors, such as ageing and obesity, as well as psychological, social, and economic components [6]. While most episodes of LBP are self-resolving, LBP can evolve into a chronic condition that has a considerable impact on the ability of patients to work, engage in social situations, and manage daily tasks and activities. CLBP represents one of the five major causes of living with disability worldwide [7].

The complexity of spinal anatomy makes it prone to a wide array of stressors that can lead to different types of LBP, such as mechanical, discogenic, or neurologic, alone or in combination [8]. In most cases, a specific nociceptive cause [6] cannot be identified, making it difficult to determine the best management course.

For patients with mechanic or non-specific cLBP, current guidelines suggest conservative, nonpharmacologic management as a first-line treatment; in particular, the available evidence lists physical exercise and passive management, such as massage, as viable options for these patients [9,10,11]. Among available strategies, physiotherapy represents a common option offered to patients presenting with cLBP, and several investigations have sought to highlight which physiotherapeutic option is the most effective in this setting [12,13,14,15,16,17]. However, this type of management can often be time-consuming and costly, requiring multiple treatment sessions over numerous weeks [18,19,20]. It is thus fundamental to identify which patients can indeed profit from this kind of management, to focus the available resources on patients who are most likely to benefit from it, and promptly initiate a different treatment for those who would probably not see any meaningful improvement after physiotherapy. Therefore, a level I systematic review was conducted to investigate whether demographic characteristics at baseline influence the outcome of patients undergoing physiotherapy for mechanic or non-specific cLBP. The outcomes of interest were to assess the association between patient characteristics (length of the follow-up, mean age and BMI, sex, and previous symptoms duration) and PROMs (VAS, RMQ, and ODI) at baseline and the same PROMs (VAS, RMQ, and ODI) at the last follow-up.

## 2. Methods

### 2.1. Eligibility Criteria

All the randomised controlled trials (RCTs) that evaluated a physiotherapy programme’s efficacy in patients with LBP were accessed. According to the authors’ language capabilities, English, German, Italian, French, and Spanish articles were eligible. According to the Oxford Centre of Evidence-Based Medicine [21], we included only RCTs with level I of evidence. Opinions, editorials, letters, and reviews were not examined. Animal, biomechanical, computational, cadaveric, and other in vitro studies were excluded. All studies evaluating non-specific [11] or mechanical [22] chronic low back pain were eligible. The pain was defined as chronic when symptoms persisted for at least three months [23]. The presence of concomitant radiculopathy, psychologic disorders, or concomitant pharmacological therapy did not warrant exclusion from the study. We excluded studies that had missing quantitative data on the outcomes of interest.

### 2.2. Search Strategy

This study was conducted according to the Preferred Reporting Items for Systematic Reviews and Meta-Analyses: the 2020 PRISMA statement [24]. The following algorithm was used for the literature search:

- Problem: LBP;
- Intervention: physiotherapy;
- Outcomes: prognostic factors.

In September 2024, the following databases were accessed: PubMed, Web of Science, Google Scholar, and Embase. No time constraint was established for the search. The search was restricted to only RCTs. No additional filters were used in the database search. The matrix of keywords used in each database was as follows: “Low Back Pain”[Mesh] OR “Low Back Pain/therapy”[Mesh] OR “Low Back Pain/rehabilitation”[Mesh] OR “Spine”[Mesh] OR Low back pain OR LBP OR non-specific Low back pain OR mechanical Low back pain OR chronic Low back pain OR spine AND “Physical Therapy Modalities”[Mesh] OR physiotherapy AND “Musculoskeletal Manipulations”[Mesh] OR “Massage”[Mesh] OR “Manipulation, Spinal”[Mesh] OR “Muscle Stretching Exercises”[Mesh] OR “Exercise”[Mesh] OR “Exercise Therapy”[Mesh] OR “Acupuncture”[Mesh] OR “Acupuncture Therapy”[Mesh] OR “Yoga”[Mesh] OR physical therapy OR manual therapy OR massage OR mobilization OR spinal manipulation OR lumbar stabilization OR active and passive stretching OR exercises OR muscle exercises OR motor control exercises OR strengthening exercises OR stabilizing exercises OR functional resistance training OR muscle strength training OR Back school OR McKenzie OR Acupuncture OR Pilates OR Yoga AND “Prognosis”[Mesh] OR prognostic factors.

### 2.3. Selection and Data Collection

Two authors (G.P. and M.P.) separately executed the database search. All the concordant titles were screened by hand, and the abstracts were analysed if suitable. The full text of the abstracts that matched the topic was accessed. Studies with an inaccessible or unavailable full text were not considered for inclusion. A cross-reference of the full-text bibliography was also conducted to identify additional studies. In cases of disagreement, a third senior author (N.M.) made the ultimate decision.

### 2.4. Data Items

Two authors (G.P.; M.-K.M.) individually conducted data extraction. The following data at baseline were extracted: the author, year and journal of publication, gender ratio, number of patients and their associated mean age and BMI (kg/m^2^), mean duration of symptom duration before physiotherapy, and period of the follow-up. Data regarding the following PROMs were acquired at baseline and at the last follow-up: the Visual Analogic Scale (VAS) or numeric rating scale (NRS), Roland Morris Disability Questionnaire (RMQ) [25], and Oswestry Disability Index (ODI) [26]. Given the high correlation between VAS and NRS, these two parameters were used interchangeably [27]. Data were extracted in Microsoft Office Excel version 16.72 (Microsoft Corporation, Redmond, WA, USA).

### 2.5. Assessment of the Risk of Bias and Quality of the Recommendations

The risk of bias in RCTs was examined in conformity with the Cochrane Handbook for Systematic Reviews of Interventions guidelines [28]. The risk of bias graph of the software Review Manager (RevMan 5.3, The Nordic Cochrane Collaboration, Copenhagen) was used. One reviewer (A.B.) autonomously evaluated the risk of bias in the reduced studies. The following endpoints were analysed: selection, detection, performance, attrition, reporting, and other biases.

### 2.6. Synthesis Methods

The main author (F.M.) performed the statistical analyses following the recommendations of the Cochrane Handbook for Systematic Reviews of Interventions [29]. For descriptive statistics, IBM SPSS version 25 was used. The mean and standard deviation were applied. A multiple pairwise analysis was performed to investigate possible associations between patient characteristics (mean follow-up, mean age, female sex, mean BMI, and symptom duration) and PROMs (VAS, RMQ, and ODI) at baseline and the same PROMs (VAS, RMQ, and ODI) at the last follow-up. The STATA Software/MP version 16 (StataCorporation, College Station, TX, USA) was used for the analyses. A multiple linear model regression analysis through the Pearson Product–Moment Correlation Coefficient (r) was used. The Cauchy–Schwarz formula was used for inequality: +1 is considered a positive linear correlation and −1 negative. Values of 0.1 < | r | < 0.3, 0.3 < | r | < 0.5, and | r | > 0.5 were considered to have weak, moderate, and strong correlations, respectively. The overall significance was assessed through a χ^2^ test, with values of *p* < 0.05 considered statistically significant.

## 3. Results

### 3.1. Study Selection

**Figure 1:**
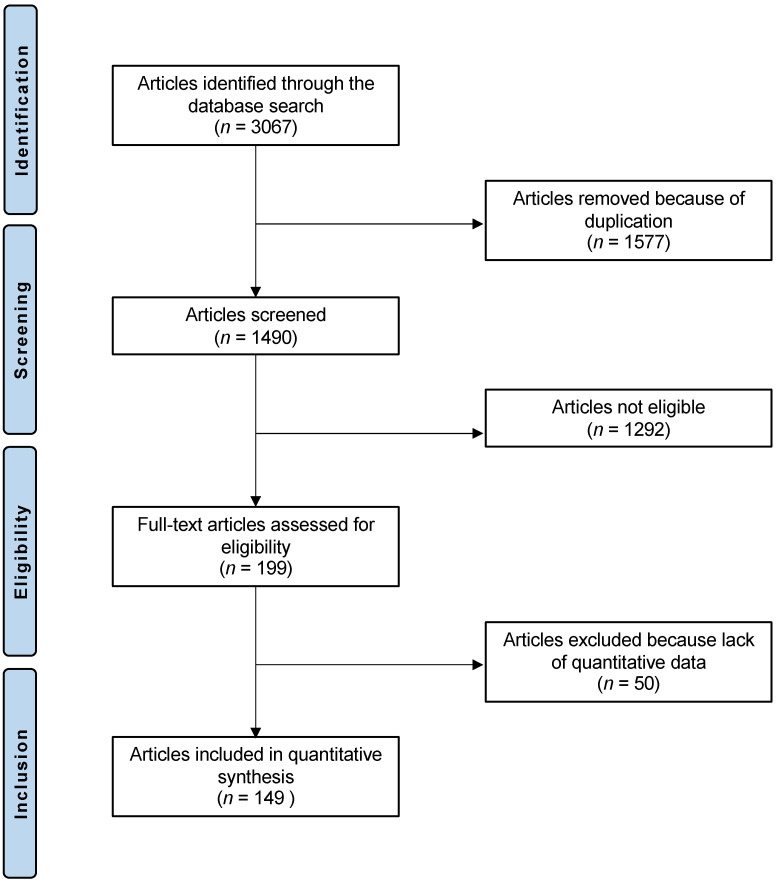
PRISMA flow chart of literature search.

The systematic literature search led to 3067 articles. Of them, a total of 1577 duplicates were excluded. Furthermore, 1292 studies did not fulfil the eligibility criteria and were therefore not included for the following reasons: study design (*n*  =  1042), low level of evidence (*n* = 128), therapeutic approaches not suitable (*n* = 69), and language limitations (*n*  =  53). An additional 50 papers were excluded because quantitative data on the interest outcomes were unavailable. Finally, 149 RCTs were included in our study. The results of the literature search are visible in Figure 1.

### 3.2. Risk of Bias Assessment

**Figure 2:**
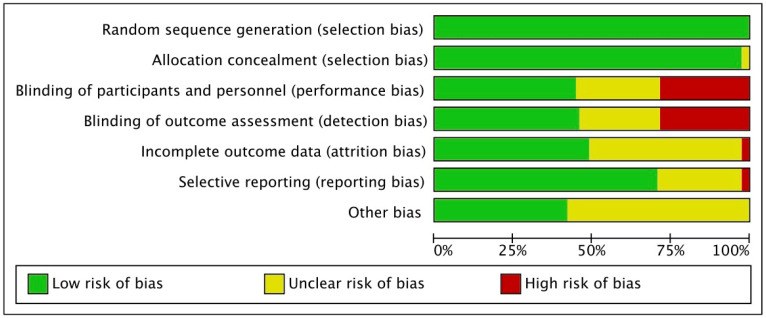
Cochrane risk of bias tool graph.

The risk of bias analysis indicated a low risk of selection bias, as all studies included were randomised controlled trials (RCTs). Most studies demonstrated high quality in terms of how patients were assigned to treatment groups, leading to a low to moderate risk of allocation bias. Given the lack of blinding of patients and personnel, 25% of the RCTs raised a high risk of performance and assessment bias. Another 25% of the included studies did not report information on blinding during the procedure and assessment of the outcomes and were judged as having a moderate risk of bias in these two domains. In certain studies, details about participant dropouts during enrolment or analysis were reported inadequately, leading to moderate attrition bias. The risk of reporting bias was generally low to moderate, while the risk of other biases was predominantly low. In conclusion, the methodological assessment of RCTs was good (Figure 2).

### 3.3. Study Characteristics and Results of Individual Studies

Data from 12,625 patients were retrieved. The mean length of symptoms before the treatment was 61.6 ± 51.2 months. The mean length of the follow-up was 4.3 ± 5.9 months. The mean age of patients was 44.4 ± 9.4 years and the mean BMI was 25.7 ± 2.5 kg/m^2^. The generalities and demographics of the included studies are shown in Table 1.

### 3.4. Synthesis of Results

VAS at the last follow-up evidenced a weak positive association with VAS at baseline (*r* = 0.2; *p* = 0.0005). RMQ at the last follow-up was moderately associated with patient age at baseline (*r* = 0.4; *p* < 0.0001) and moderately associated with RMQ at baseline (*r* = 0.4; *p* < 0.0001). ODI at the last follow-up evidenced a weak positive association with patient age at baseline (*r* = 0.2; *p* = 0.04). In addition, ODI at the last follow-up was moderately associated with BMI at baseline (*r* = 0.4; *p* = 0.0001) and strongly associated with ODI at baseline (*r* = 0.6; *p* < 0.0001). No additional significant associations were evidenced (Table 2).

**Table 1:** Generalities and patient baseline of the included studies (RCT: randomised controlled trial).

Author, Year	Journal	Type of Treatment	Type of Movement	Patients (n)	Follow-Up (Months)	Mean Age	Women (%)
Aasa et al., 2015 [30]	*J Orthop Sports Phys Ther*	Exercise	Low-Load	25	12	42.0	54
		Exercise	High-Load-Lifting	28		42.0	57
Balthazard et al., 2012 [31]	*BMC Musculoskelet Disord*	Spinal Manipulation	High-Velocity, Low-Amplitude	19	6	44.0	36
		Physical Agents	Ultrasound	18		42.0	30
Bhadauria et al., 2017 [32]	*J Exerc Rehabil*	Exercise	Stabilisation	12	0	32.8	50
		Exercise	Strengthening	12		36.7	42
		Pilates	Contraction	12		35.3	8
Cecchi et al., 2010 [33]	*Clin Rehabil*	Back School	Individualised	68	12	57.9	70
		Physiotherapy	Individualised	68		60.5	61
		Spinal Manipulation	Mobilisation, Manipulation	69		58.1	69
Costa et al., 2009 [34]	*Phys Ther*	Motor Control Exercise	Individualised	77	10	54.6	58
		Sham		77		52.8	62
Vibe Fersum et al., 2019 [35]	*Eur J Pain*	Spinal Manipulation	Individualised	59	36	43.1	52
		Cognitive Functional Therapy		62		42.9	53
Garcia et al., 2013 [36]	*Phys Ther*	Back School	Unknown	74	6	54.2	69
		Mckenzie	Symptom-Guided	74		53.7	78
Goldby et al., 2006 [37]	*Spine*	Spinal Stabilisation	Stabilisation	35	24	43.4	68
		Spinal Manipulation	Individualised	37		41.0	70
		Control		19		41.5	68
Halliday et al., 2016 [38]	*J Orthop Sports Phys Ther*	Mckenzie	Symptom-Guided	32	2	48.8	80
		Motor Control Exercise	Contraction	30		48.3	80
Hohmann et al., 2018 [39]	*Dtsch Arztebl Int*	Hirudotherapy		25	2	59.3	88
		Exercise	Various	19	1	56.5	95
Kääpä et al., 2006 [40]	*Spine*	Multidisciplinary	Various	59	24	46.0	98
		Physiotherapy	Various	61		46.5	
Kobayashi et al., 2019 [41]	*Complement Ther Med*	Shiatsu	Various	30	2	67.4	67
		Standard Care		29		68.3	62
Lawand et al., 2015 [42]	*Joint Bone Spine*	Exercise	Stretching	30	6	49.4	81
		Control		30		47.5	73
Macedo et al., 2019 [43]	*Physiotherapy*	Kinesio Taping with Tension	Traction	27	0.33	25.0	100
		Kinesio Taping no Tension		27		24.0	
		Sham		27		25.0	
		Control		27		24.0	
Majchrzycki et al., 2014 [44]	*Sci World J*	Massage	Deep Tissue Massage	28	0	52.6	46
		Massage	Deep Tissue Massage	26		50.8	50
Murtezani et al., 2015 [45]	*J Back Musculoskelet Rehabil*	Mckenzie	Symptom Guided	110	3	48.8	25
		Physical Agents	Various	109		47.5	62
Sahin et al., 2017 [46]	*Turk J Phys Med Rehab*	Physical Agents	Various	50	12	50.4	64
		Control	Stretching And Strengthening	50		46.2	62
Saper et al., 2017 [47]	*Ann Intern Med*	Yoga	Various	127	9.2	46.4	57
		Aerobics	Various	129		46.4	70
		Education		64		44.2	66
Suh et al., 2019 [48]	*Medicine*	Stretching	Stretching	13	1.5	53.5	62
		Walking Exercise	Walking	13		54.2	85
		Spinal Stabilisation	Stabilisation	10		57.4	60
		Spinal Stabilisation	Stabilisation, Walking	12		54.8	67
Takahashi et al., 2017 [49]	*Fukushima J Med Sci*	Control		15	0	53.3	53
		Exercise	Stretching And Strengthening	18		57.6	56
Uzunkulaoğlu et al., 2018 [50]	*Turk J Phys Med Rehabil*	Kinesio Taping with Tension	Traction	30	6	21.6	63
		Kinesio Taping without Tension		30		21.3	63
Yeung et al., 2003 [51]	*J Altern Complement Med*	Exercise	Various	26	3	55.6	81
		Exercise	Various	26		50.4	85
Dufour et al., 2010 [52]	*Spine*	Exercise	Strengthening	129	24	41.2	57
		Exercise	Strengthening	143		40.6	56
Helmhout et al., 2004 [53]	*Spine*	Exercise	High-Intensity Strengthening	41	9	41.0	0
		Exercise	Low-Intensity Strengthening	40		40.0	
Jarzem et al., 2005 [54]	*J Musculoskelet Pain*	Sham		83	1	45.1	50
		Tens		84			
		Acupuncture TENS		78			
		Tens	Biphasic	79			
Meng et al., 2011 [55]	*Clin J Pain*	Back School		181	12	50.2	65
		Back School		163		49.5	63
Prommanon et al., 2015 [56]	*J Phys Ther Sci*	Back Care Pillow		26	3	38.5	42
		Control		26		39.7	50
Tavafian et al., 2011 [57]	*Clin J Pain*	Education		97	6	44.6	73
		Control		100		45.9	83
Tavafian et al., 2014 [58]	*Int J Rheum Dis*	Education		87	12	44.6	75
		Control		91		46.2	82
Alfuth et al., 2016 [59]	*Orthopäde*	Mobilisation	Mobilisation	14	1	50.0	79
		Stabilisation	Stabilisation	13		43.0	54
Grande-Alonso et al., 2019 [60]	*Pain Med*	Multidisciplinary	Various	25	3	39.9	56
		Multidisciplinary	Stabilisation	25		38.3	56
Ali et al., 2019 [61]	*J Bodyw Mov Ther*	Spinal Manipulation	Various	14	0	35.4	
		Spinal Manipulation	Various	14		35.3	
Ahmadi et al., 2020 [62]	*Clin Rehabil*	Exercise	Individualised	30	0	42.6	100
		Education	Individualised	29		38.9	100
Almhdawi et al., 2020 [63]	*Clin Rehabil*	Exercise	Strengthening, Stretching	21	0	40.5	34
		Control		20		41.7	20
Added et al., 2016 [64]	*J Orthop Sports Phys Ther*	Physiotherapy	Individualised	74	6	44.6	72
		Physiotherapy	Individualised	74	6	45.6	72
Arampatzis et al., 2017 [65]	*Eur J Appl Physiol*	Exercise	Low–moderate Intensity	20	0	31.9	40
		Control		20		31.4	45
Areeudomwong et al., 2016 [66]	*Musculoskeletal Care*	Exercise	Contraction	21	3	35.4	71
		Control		21		36.2	76
Bae et al., 2018 [67]	*J Back Musculoskelet Rehabil*	Exercise	Core Stabilisation	18	3	32.7	50
		Exercise	Strengthening	18		32.4	61
Bi et al., 2013 [68]	*Int J Med Res*	Exercise	Contraction	23	0	29.1	44
		Control	Strengthening	24		30.9	46
Bicalho et al., 2010 [69]	*Man Ther*	Manipulation	High-Velocity	20	0	29.5	75
		Control		20		26.5	60
Bronfort et al., 2011 [70]	*Spine J*	Exercise	Various	101	9	45.6	58
		Spinal Manipulation	High-Velocity, Low-Amplitude	100		45.2	66
		Exercise	Strengthening	100		44.5	57
Cai et al., 2017 [71]	*Med Sci Sports Exerc*	Exercise	Resistance	25	4	28.9	50
		Exercise	Contraction	24		26.1	
		Exercise	Stabilisation	25		26.9	
Azevedo et al., 2017 [72]	*Phys Ther*	Exercise	Strengthening, Stretching	74	4	40.4	58
		Control	Various	74		43.4	65
Castro-Sánchez et al., 2016 [73]	*Spine J*	Spinal Manipulation	High-Velocity	31	0.25	43.0	65
		Spinal Manipulation	Low-Velocity	31		47.0	61
Ryan et al., 2010 [74]	*Man Ther*	Exercise	Various	20	3	45.2	70
		Control		18		45.5	61
Chhabra et al., 2018 [75]	*Eur Spine J*	App	Various	45	0	41.4	
		Control		48		41.0	
Cortell-Tormo et al., 2018 [76]	*J Back Musculoskelet Rehabil*	Exercise	Various	11	0	35.6	100
		Control		8		35.6	100
Cruz-Díaz et al., 2015 [77]	*Disabil Rehabil*	Pilates	Individualised	53	10.5	69.6	100
		Physiotherapy	Various	48		72.7	100
Cruz-Díaz et al., 2017 [78]	*Complement Ther Med*	Pilates	Various	34	0	36.9	68
		Pilates	Various	34		35.5	62
		Control		30		36.3	63
Cuesta-Vargas et al., 2011 [79]	*Am J Phys Med Rehabil*	Multidisciplinary	Various	24	0	37.6	58
		Multidisciplinary	Various	25		39.8	54
Diab et al., 2013 [80]	*J Back Musculoskelet Rehabil*	Exercise	Traction	40	6	46.3	45
		Exercise	Stretching	40		45.9	43
Koldaş Doğan et al., 2008 [81]	*Clin Rheumatol*	Aerobics	Walking	19	1	37.1	79
		Physical Agents	Various	18		41.5	78
		Control		18		42.1	78
Eardley et al., 2013 [82]	*Forsch Komplementmed*	Multidisciplinary	Individualised	20	0	48.8	85
		Sham	Individualised	21		48.1	67
		Delayed		17		44.6	65
Engbert et al., 2011 [83]	*Spine*	Exercise	Climbing	10	0	51.9	60
		Exercise	Various	13		50.4	46
de Oliveira et al., 2013 [84]	*Phys Ther*	Spinal Manipulation	High-Velocity	74	0	46.0	68
		Spinal Manipulation	Region-Specific	74		46.3	80
França et al., 2012 [85]	*J Manipulative Physiol Ther*	Exercise	Stabilisation	15	0	42.1	
		Exercise	Stretching	15		41.5	
Friedrich et al., 1998 [86]	*Arch Phys Med Rehabil*	Exercise	Various	44	12	43.3	57
		Exercise	Various	49		44.9	45
Frost et al., 1995 [87]	*BMJ*	Exercise	Aerobics	36	6	34.2	53
		Control		35		38.5	51
Garcia et al., 2018 [88]	*BMJ*	Mckenzie	Various	74	10.75	57.5	78
		Control		73		55.5	74
Gardner et al., 2019 [89]	*BMJ*	Exercise	Individualised	37	10	44.0	66
		Control	Various	38		45.0	49
Gavish et al., 2015 [90]	*Physiotherapy*	Exercise	Oscillation	18	0.75	53.2	33
		Control		18		47.1	56
Geisser et al., 2005 [91]	*Clin J Pain*	Exercise	Various	21	0	39.3	67
		Exercise	Various	18		38.7	56
		Non-Specific Exercise	Various	15		36.5	80
		Non-Specific Exercise	Various	18		46.3	61
Gwon et al., 2020 [92]	*Physiother Theory Pract*	Exercise	Side Bridge	15	0	21.9	2
		Exercise	Side Bridge	15		21.6	2
Haas et al., 2014 [93]	*Spine J*	Sham	Various	95	10.6	40.9	49
		Spinal Manipulation	High-Velocity, Low-Amplitude	99		41.4	49
		Spinal Manipulation	High-Velocity, Low-Amplitude	97		41.8	49
		Spinal Manipulation	High-Velocity, Low-Amplitude	100		41.2	52
Halliday et al., 2019 [94]	*Physiotherapy*	Mckenzie	Symptom-Guided	35	10	48.8	80
		Motor Control Exercise	Contraction	35		48.3	80
Harts et al., 2008 [95]	*Aust J Physiother*	Exercise	High-Intensity	23	4	44.0	0
		Exercise	Low-Intensity	21		42.0	
		Control		21		41.0	
Macedo et al., 2014 [96]	*Phys Ther*	Exercise	Various	86	12	49.6	52
		Motor Control Exercise	Symptom-Guided	86		48.7	66
Javadian et al., 2012 [97]	*J Back Musculoskelet Rehabil*	Exercise	Stabilisation	30	3		
		Exercise	Various				
Loss et al., 2020 [98]	*Chiropr Man Ther*	Spinal Manipulation	Thrust	12	0	41.7	50
		Control	Various	12		43.9	50
Kell et al., 2011 [99]	*J Strength Cond Res*	Exercise	Strengthening	60	0.25	42.4	31
		Exercise	Strengthening	60		41.7	37
		Exercise	Strengthening	60		42.8	33
		Control		60		43.2	38
Kim et al., 2015 [100]	*Clin Rehabil*	Exercise	Contraction	27	2	29.7	100
		Control		26		28.6	
Kim et al., 2018 [101]	*J Sport Rehabil*	Exercise	Various	38	3	39.5	61
		Exercise	Stabilisation	39		46.2	54
Tekur et al., 2008 [102]	*J Altern Complement Med*	Yoga	Various	40	0	49.0	53
		Exercise	Various	40		48.0	38
Tekur et al., 2012 [103]	*Complement Ther Med*	Yoga	Various	40	0	49.0	53
		Exercise	Various	40		48.0	38
de Oliveira et al., 2020 [104]	*J Physiother*	Spinal Manipulation	High-Velocity	71	5	45.0	77
		Spinal Manipulation	Various	72		45.0	78
Zou et al., 2019 [105]	*Medicina*	Tai Chi	Various	15	0	58.1	73
		Exercise	Stabilisation	15		58.4	73
		Control		13		60.7	77
Zhang et al., 2014 [106]	*J Int Med Res*	Education	Strengthening	25	0	22.3	33
		Control	Strengthening	24		23.0	41
Zheng et al., 2012 [107]	*J Tradit Chin Med*	Massage	Pressure; Traction	30	0	43.0	44
		Control	Traction	30		42.0	50
Yang et al., 2021 [108]	*J Bodyw Mov Ther*	Pilates	Various	20	4.5	50.5	75
		Control		19		47.9	79
Waseem et al., 2019 [109]	*J Back Musculoskelet Rehabil*	Exercise	Core Stabilisation	53	0	46.4	34
		Exercise	Various	55		45.5	35
Williams et al., 2005 [110]	*Pain*	Yoga	Various	20	0	48.7	65
		Control		24		48.0	71
Verbrugghe et al., 2021 [111]	*Int J Environ Res Public Health*	Exercise	High-Intensity Strengthening	16	6	44.3	68
		Control	Moderate-Intensity Strengthening	13		44.0	68
Sipaviciene et al., 2020 [112]	*Clin Biomech*	Exercise	Stabilisation	35	3	38.3	100
		Exercise	Strengthening	35		38.5	100
Phattharasupharerk et al., 2018 [113]	*J Bodyw Mov Ther*	Qi Gong	Various	36	0	35.7	67
		Control		36		34.8	61
Magalhães et al., 2018 [114]	*Braz J Phys Ther*	Exercise	Various	33	0	46.6	76
		Exercise	Various	33		47.2	73
Monticone et al., 2013 [115]	*Clin J Pain*	Exercise	Various	45	12	49.0	60
		Control	Various	45		49.7	56
Monticone et al., 2014 [116]	*Eur Spine J*	Motor Control Exercise	Stabilising	10	3	58.9	70
		Control	Various	10		56.6	40
Morone et al., 2011 [117]	*Eur J Phys Rehabil Med*	Back School	Various	41	5	61.2	59
		Control		29		58.6	72
Matarán-Peñarrocha et al., 2020 [118]	*Clin Rehabil*	Exercise	Various	32	6	54.3	53
		Exercise		32		53.2	47
Laosee et al., 2020 [119]	*Complement Ther Med*	Massage	Pressure	70	3.45	68.2	77
		Massage	Pressure	70		69.1	71
Rittweger et al., 2002 [120]	*Spine*	Exercise	Extension	25	6	49.8	44
		Exercise	Vibration	25		54.1	52
Prado et al., 2019 [121]	*Physiother Theory Pract*	Exercise	Stretching	27	0	35.0	70
		Control		27		33.0	63
Vollenbroek-Hutten et al., 2004 [122]	*Clin Rehabil*	Multidisciplinary	Various	69	6	38.5	
		Control		73		39.5	
del Pozo-Cruz et al., 2011 [123]	*J Rehabil Med*	Exercise	Vibration	25	0	58.7	74
		Control		24		59.5	72
Kostadinovic et al., 2020 [124]	*J Back Musculoskelet Rehabil*	Exercise	Stabilisation; Mobilisation	40	0	44.1	55
		Exercise	Stabilisation	40		44.3	58
Monticone et al., 2016 [125]	*Eur J Pain*	Exercise	Individualised	75	24	53.2	63
		Control		75		53.8	60
Járomi et al., 2018 [126]	*J Clin Nurs*	Back School	Various	67	0	41.7	94
		Control		70		41.1	93
Liu et al., 2019 [127]	*Int J Environ Res Public Health*	Tai Chi	Various	15	0	58.1	73
		Exercise	Stabilisation	15		58.4	73
		Control		13		60.7	77
Lara-Palomo et al., 2012 [128]	*Clin Rehabil*	Massage	Interferential Current	30	0	50.0	70
		Massage	Superficial Pressure	31		47.0	65
Saha et al., 2019 [129]	*Complement Ther Clin Pract*	Massage	Pressure	25	0.71	52.2	68
		Control		25		47.2	88
Segal-Snir et al., 2016 [130]	*J Back Musculoskelet Rehabil*	Exercise	Rotation	20	1	57.2	100
		Control		15		54.7	100
Nambi et al., 2014 [131]	*Int J Yoga*	Yoga	Various	30	6	44.3	63
		Control	Strengthening, Stretching	30		43.7	43
Salamat et al., 2017 [132]	*J Bodyw Mov Ther*	Exercise	Stabilisation	12	0	35.8	
		Motor Control Exercise	Various	12		36.1	
Salavati et al., 2015 [133]	*J Bodyw Mov Ther*	Exercise	Stabilisation	20	0	32.6	0
		Control	Various	20		29.9	0
Masharawi et al., 2013 [134]	*J Back Musculoskelet Rehabil*	Exercise	Various	20	2	52.5	100
		Control		20		53.6	100
Natour et al., 2014 [135]	*Clin Rehabil*	Pilates	Various	30	2.96	47.8	80
		Control		30		48.1	77
Murtezani et al., 2011 [136]	*Eur J Phys Rehabil Med*	Exercise	Individualised	50	0	51.4	48
		Control	Various	51			49
Kogure et al., 2015 [137]	*PLoS One*	Spinal Manipulation	Various	90	6	60.0	60
		Sham	Various	89		59.6	64
Ozsoy et al., 2019 [138]	*Dove Med Press*	Exercise	Core Stabilisation	21	0	68.1	29
		Exercise	Various	21		68.0	31
Jousset et al., 2004 [139]	*Spine*	Multidisciplinary	Various	43	4.75	41.4	30
		Exercise	Individualised	41		39.4	37
Roche-Leboucher et al., 2011 [140]	*Spine*	Exercise	Various	68	12	40.8	32
		Exercise	Various	64		38.7	38
Khalil et al., 1992 [141]	*Spine*	Spinal Manipulation	Stretching	14	0	41.1	43
		Control		14		48.5	50
Mannion et al., 1999 [142]	*Spine*	Exercise	Various	46	6	46.3	61
		Aerobics	Low-Impact	47		45.2	54
		Physical Agents	Various	44		43.7	55
Mannion et al., 2001 [143]	*Spine*	Exercise	Various	44	12	46.3	61
		Aerobics	Low-Impact	43		45.2	54
		Physical Agents	Various	40		43.7	55
Yoon et al., 2012 [144]	*Ann Rehabil Med*	Massage	Symptom-Guided	12	0.5	50.3	58
		Tens	Various	10		53.3	60
Yang et al., 2019 [145]	*J Healthc Eng*	Exercise	Symptom-Guided	5	0	35.0	20
		Control	Various	3		50.3	100
Hicks et al., 2016 [146]	*Clin J Pain*	Control	Various	31	3	69.5	52
		Exercise	Stabilisation	26		70.7	58
Yalfani et al., 2020 [147]	*J Bodyw Mov Ther*	Water Pilates	Various	12	0	25.2	100
		Pilates	Various	12		24.7	100
Trapp et al., 2015 [148]	*J Back Musculoskelet Rehabil*	Exercise	Feedback	15	0	45.5	33
		Control	Various	15		40.6	40
Kofotolis et al., 2016 [149]	*J Back Musculoskelet Rehabil*	Pilates	Various	37	0	42.7	100
		Control		28		41.2	100
		Exercise	Strengthening	36		39.1	100
Kuvacic et al., 2018 [150]	*Complement Ther Clin Pract*	Control		15	0	33.6	53
		Yoga	Various	15		34.7	40
Hernandez-Reif et al., 2001 [151]	*Intern J Neuroscience*	Massage	Various	24	0	43.8	58
		Control	Various			36.7	50
Lewis et al., 2005 [152]	*Spine*	Exercise	Various	33	12	46.1	65
		Exercise	Individualised	29		45.7	65
O’Keeffe et al., 2020 [153]	*J Sports Med*	Cognitive Functional Therapy	Individualised	106	10 to 10.5	47.0	77
		Exercise	Various	100		50.6	70
Kaeding et al., 2017 [154]	*Scand J Med Sci Sports*	Exercise	Vibration	21	0	46.4	67
		Control		20		44.6	70
Petrozzi et al., 2019 [155]	*Chiropr Man Therap*	App	Various	54	max 10	50.1	54
		Control	Various	54		50.6	59
Winter et al., 2015 [156]	*J Back Musculoskelet Rehabil*	Exercise	Rotation	10	0	45.9	45
		Exercise	Stretching	10		48.9	
		Exercise	Strengthening	10		38.3	
Massé-Alarie et al., 2016 [157]	*Clin Neurophysiol*	Physical Agents	Contraction	11	0	33.2	45
		Sham	None	10		42.1	50
Martí-Salvador et al., 2018 [158]	*Arch Phys Med Rehabil*	Spinal Manipulation	Various	33	2	43.4	52
		Sham	Various	33		41.7	61
Aguilar-Ferrándiz et al., 2022 [159]	*Nature*	Kinesio Taping	Without Tension	29	0	44.0	59
		Tens		29		46.0	72
Elgendy et al., 2022 [160]	*Ortop Traumatol Rehabil*	Physical Agents	Various	15	0	32.7	
		Control	Stretching, Strengthening	15		33.3	
Fukuda et al., 2021 [161]	*Braz J Phys Ther*	Spinal Manipulation	Joint Mobilisation	35	12	35.2	53
		Spinal Manipulation	Joint Mobilisation, Strengthening	35		40.2	
Ma et al., 2021 [162]	*Ann Palliat Med*	Physical Agents	Needling	30	12	47.7	50
		Massage	Swedish Massage	30		49.2	63
Maggi et al., 2022 [163]	*Aging Clin Exp Res*	Kinesio Taping	No Tension	57	3	66.8	72
		Control		62		67.8	82
Jalalvandi et al., 2022 [164]	*BMC Musculoskelet Disord*	Exercise	Stretching, Strengthening	22	0	37.9	73
		Tens		22		36.1	64
Atilgan et al., 2021 [165]	*J Back Musculoskelet Rehabil*	Exercise	Breathing, Stabilisation	23	0	32.1	100
		Control	Stabilisation	20		37.7	100
Pivovarsky et al., 2021 [166]	*Einstein (Sao Paulo)*	Sham		35	0	40.8	69
		Tens		35		44.0	66
		Tens		35		42.6	77
Van Dillen et al., 2021 [167]	*JAMA Neurol*	Exercise	Various	74	12	42.4	68
		Exercise	Stretching, Strengthening	75		42.6	55
Vibe Fersum et al., 2013 [168]	*Eur J Pain*	Spinal Manipulation	Joint Mobilisation	43	0	42.9	49
		Cognitive Functional Therapy	Unknown	51		41.0	53
Ghroubi et al., 2007 [169]	*Ann Readapt Med Phys*	Spinal Manipulation	Symptom-Guided	32	1	39.1	84
		Sham		32		37.4	75
Huber et al., 2019 [170]	*BMC Musculoskelet Disord*	Walking	Walking	27	13.5	52.9	52
		Walking	Walking, Heat	26		53.4	54
		Control		27		43.8	63
Werners et al., 1999 [171]	*Spine*	Tens		74	3	38.3	43
		Massage	Traction	73		39.2	49
Kankaanpää et al., 1999 [172]	*Spine*	Exercise	Various	30	12	39.8	37
		Control	Various	24		39.3	33
Marshall et al., 2008 [173]	*Spine*	Spinal Manipulation	High-Velocity, Low-Amplitude, Various	12	9	34.3	50
		Spinal Manipulation	High-Velocity, Low-Amplitude	13		35.8	54
		Spinal Manipulation	Non-Thrust, Various	12		33.9	50
		Spinal Manipulation	Non-Thrust	13		41.7	42
Branchini et al., 2015 [174]	*F1000research*	Spinal Manipulation	Pressure	11	3	48.0	64
		Control	Individualised	13		44.0	69
Batıbay et al., 2021 [175]	*J Orthop Sci*	Pilates	Various	28	0	49.3	100
		Exercise		25		48.4	100
Elabd et al., 2020 [176]	*J Appl Biomech*	Exercise	Stabilisation, Stretching	25	0	26.8	52
		Exercise	Stabilisation	25		27.4	
Dadarkhah et al., 2021 [177]	*J Natl Med Assoc*	Exercise	Core Stabilisation	28	12	49.0	57
		Exercise	Core Stabilisation	28		50.0	57
Nardin et al., 2022 [178]	*Lasers Med Sci*	Exercise	Aerobics	20	0.5	42.2	80
		Sham	Aerobics	20		42.8	75
		Physical Agents		20		43.1	80

## 4. Discussion

Based on the main findings of this study, age and BMI might exert a limited influence on the outcomes of the physiotherapeutic management of cLBP. Pain and disability at baseline might represent important predictors of health-related quality of life at the 6-month follow-up.

Regarding demographic data, age showed a weak to moderate association with health-related quality of life (HRQoL) parameters. BMI did not show any significant correlation to any of the outcomes of interest for patients affected by cLBP. A possible explanation might lie in the relatively narrow range of age and BMI obtained from the available studies. Overall, the available literature analysing prognostic factors for physiotherapy outcomes in cLBP patients showed contrasting results for age [179,180], while BMI is often not considered in other studies. When age did show a correlation with pain and disability after physiotherapeutic management, older age was described as a negative prognostic factor [179], similar to the results obtained in the present work.

Considering the worldwide trends of obesity and the ageing population [181,182], future research should target these outliers to identify possible limits to the efficacy of physical therapy in these particularly complex subjects, which are frequently affected by cLBP [183].

Overall, pain and disability outcomes correlated with the respective baseline parameters. One possible interpretation for this result is that, since the follow-up HRQoL parameters correlate with the results at follow-up, physiotherapeutic management should be started as early as possible. Beginning the treatment with a low level of pain and disability would then be associated with lower levels of pain and disability at follow-up. A previously published secondary analysis of RCTs investigating prognostic factors for symptom improvement in patients with cLBP showed comparable results regarding the association between baseline and follow-up parameters, which were also evaluated at the 6-month mark [184]. Similar results have been observed in a surgical cohort at a 2-year follow-up [185]. While the two considered patient groups and management options are not comparable, it is possible that long-lasting pain would lead to a harder-to-manage pain memory [186], and early intervention would thus be beneficial for these patients.

The literature has reported an initial improvement for all patients with cLBP, which then slows down over time, irrespective of the type of treatment initiated [187,188]. However, the short 6-month follow-up available in the literature may not be sufficient to develop the benefits of conservative treatment fully, and a longer follow-up might be required to observe a more relevant improvement in patients with higher baseline levels of pain and disability.

The interpretation of the correlation between baseline and follow-up HRQoL must consider the short follow-up available. When analysing longitudinal data, it is crucial to consider that the shorter the interval between measurements, the stronger the correlation between the values will be. One study on the prognostic factors of cLBP with a one-year follow-up showed a relatively low effect of baseline pain and disability on the variance in HRQoL at the last follow-up [189]. Thus, a question remains regarding whether the observed correlation is a true biological association or arises from the closeness of the considered intervals. Another non-invasive option in the management of cLBP is represented by a multidisciplinary biopsychosocial model of rehabilitation, which is demonstrated to alleviate physiological, psychological, and social disabilities and ensure the return to work of patients with chronic LBP [190,191,192,193]. Another commonly used component of multidisciplinary pain programmes is represented by cognitive behavioural therapy (CBT), whose target is to replace maladaptive patient coping skills, thoughts, emotions, and behaviours with more adaptive ones. This can decrease distress, consequentially reducing the pain experience [194,195,196]. Yoga, home exercises, and Pilates also improved the outcomes of patients suffering from cLBP [197,198,199]. Ot et al. [199] randomised 54 patients with cLBP into three groups undergoing physical therapy, home exercises, and yoga. All groups showed decreased stress, pain intensity, pain sensitivity, and central sensitisation, and improved quality of life [199]. The main limitation of the included evidence is represented by the small ranges of age and BMI available in the works published so far. This restricts the generalizability of the observed results to a small population segment: further studies will be required to investigate the effects of physiotherapy on cLBP in older patients with a higher BMI. The available literature only evaluated a short follow-up, which does not allow us to infer the prognostic factors of managing cLBP over a long period. The present analysis did not allow for the differentiation of possible prognostic factors in specific patient groups, such as those with and without concomitant radiculopathy or psychologic disorders, or who those who had undergone previous surgery. Different patient categories might respond differently to physiotherapeutic management. Another aspect that should be considered in future studies is the barriers to adherence highlighted in previous studies, which could affect the long-term outcomes of physiotherapeutic management [200]. Chronic low back pain could also lead to long-term disability associated with financial damage from decreased work hours and frequent healthcare service use [201,202]. Moreover, cLBP has a significant negative psychological impact on patients, increasing the prevalence of depression, anxiety, and poor sleep quality if compared with healthy people [203,204]. A decrease in depressive symptoms is associated with a reduction in pain and improvements in function in patients with cLBP treated with physiotherapy [205,206,207]. Given the lack of sufficient quantitative data, it was not possible to evaluate the importance of psychological or occupational factors, which are known prognostic factors for the success of cLBP management [208,209,210]. Similarly, the effect of other concomitant therapies, such as pharmacological management or patient outcomes with previous guideline-nonconcordant care [211], could not be evaluated. In light of the limitations of the present study, our results should be interpreted cautiously.

## 5. Conclusions

Age and BMI might exert a limited influence on the outcomes of the physiotherapeutic management of cLBP. Moreover, pain and disability at baseline might represent important predictors of health-related quality of life at the 6-month follow-up. Further studies on a larger population with a longer follow-up are required to validate these results.

## Figures and Tables

**Table 2:** Results of the pairwise correlations.

Endpoint	VAS		RMQ		ODI	
	*r*	*p*	*r*	*p*	*r*	*p*
Follow-up (*months*)	0.0	0.7	0.0	0.7	0.0	0.7
Mean age	0.1	0.2	0.4	<0.0001	0.2	0.04
Women (*%*)	0.1	0.3	0.2	0.073	−0.1	0.3
Mean BMI	0.1	0.4	−0.1	0.6	0.4	0.0001
Symptom duration (*months*)	0.0	0.7	−0.1	0.6	0.1	0.5
VAS (*baseline*)	0.2	0.0005	0.1	0.5	0.0	0.8
RMQ (*baseline*)	0.2	0.1	0.9	<0.0001	0.5	0.02
ODI (*baseline*)	−0.1	0.2	0.6	0.003	0.6	<0.0001

## Data Availability

The datasets generated and/or analysed during the current study are available throughout the manuscript.
